# Trends and predictors of antimicrobial resistance among patients with urinary tract infections at a tertiary hospital facility in Alexandria, Egypt: a retrospective record-based classification and regression tree analysis

**DOI:** 10.1186/s12879-024-09086-6

**Published:** 2024-02-22

**Authors:** Marian Shaker, Adel Zaki, Sara Lofty Asser, Iman El Sayed

**Affiliations:** 1 Department of Biomedical Informatics and Medical Statistics, Medical Research Institute, Alexandria University, Alexandria, Egypt; 2 Department of Medical Microbiology and Immunology, Faculty of Medicine, Alexandria University, Alexandria, Egypt

**Keywords:** Urinary tract infection, Drug resistance, Microbial, Risk factors, Multiple drug-resistant, Bacteria

## Abstract

**Background:**

The incidence of Antimicrobial Resistance (AMR) in uropathogens varies between countries and over time. We aim to study the patterns and potential predictors of AMR among patients with UTIs admitted to the Urology Department at Alexandria University Hospital.

**Methods:**

An observational retrospective record-based study was conducted on all patients admitted to the Urology department from October 2018 to October 2020. Data collected from patients’ records included: demographic data, diagnosis on admission, history of chronic diseases, duration of hospital stay, insertion of a urinary catheter, duration of the catheter in days, history of the use of antibiotics in the previous three months, and history of urinary tract operations. If UTI was documented, we abstracted data about urine culture, use of antibiotics, results of urine cultures, type of organism isolated, and sensitivity to antibiotics. We conducted a multivariable logistic regression model. We performed Classification and Regression Tree Analysis (CART) for predicting risk factors associated with drug resistance among patients with UTI. Data were analyzed using SPSS statistical package, Version 28.0, and R software (2022).

**Results:**

This study encompassed 469 patients with UTIs. The most commonly isolated bacterium was *Escherichia coli*, followed by *Klebsiella pneumoniae*. Multidrug resistance (MDR) was found in 67.7% (149/220) of patients with hospital-acquired UTIs and in 49.4% (83/168) of patients with community-acquired UTIs. Risk factors independently associated with antimicrobial resistance according to logistic regression analysis were the use of antibiotics within three months (AOR = 5.2, 95% CI 2.19–12.31), hospital-acquired UTI (AOR = 5.7, 95% CI 3.06–10.76), diabetes mellitus (AOR = 3.8, 95% CI 1.24–11.84), age over 60 years (AOR = 2.9, 95% CI 1.27–6.72), and recurrent UTI (AOR = 2.6, 95% CI 1.08–6.20). Classification and regression tree (CART) analysis revealed that antibiotic use in the previous three months was the most significant predictor for developing drug resistance.

**Conclusion:**

The study concluded a high level of antimicrobial resistance as well as significant MDR predictors among hospitalized patients with UTIs. It is vital to assess resistance patterns in our hospitals frequently to improve rational antibiotic treatment as well as to sustain antimicrobial stewardship programs and a rational strategy in the use of antibiotics. Empirical therapy for UTI treatment should be tailored to the potential pathogens’ susceptibility to ensure optimal treatment. Strategic antibiotic use is essential to prevent further AMR increases. Further research should focus on suggesting new biological systems or designed drugs to combat the resistance of UTI pathogens.

**Supplementary Information:**

The online version contains supplementary material available at 10.1186/s12879-024-09086-6.

## Introduction

Urinary tract infection (UTI) is the second most prevalent community and hospital acquired bacterial disease after respiratory tract infections (RTIs) [1]. Antimicrobial resistance (AMR) is a global public health crisis that threatens our ability to successfully treat infections [2]. According to the World Health Organization (WHO) definition, AMR develops when bacteria, viruses, fungi, and parasites adapt over time and stop responding to medications, making illnesses harder to cure and increasing the risk of disease spreading, serious illness, and death [3]. AMR causes extended patient morbidity and mortality [4]. Increasing AMR has transcended hospital boundaries and impacted individuals with community-acquired and hospital-acquired urinary tract infections (UTIs) [5]. Globally, approximately 700,000 deaths are attributed annually to AMR, and this number could increase to 10 million deaths per year by 2050 [6]. 

Multidrug-resistant (MDR), extensively drug-resistant (XDR), and pan drug-resistant (PDR) bacteria are frequently used in medical literature to characterize different resistance patterns found in AMR bacteria. MDR was defined as acquired non-susceptibility to at least one agent in three or more antimicrobial categories, XDR was defined as non-susceptibility to at least one agent in all but two or fewer antimicrobial categories (i.e., bacterial isolates remain susceptible to only one or two categories), and PDR was defined as non-susceptibility to all agents in all antimicrobial categories [7]. 

The risk factors of multidrug resistant organism (MDRO)–induced UTIs can be categorized as demographic factors—old age, female sex—and individual factors—a history of UTIs, a dementia or malfunction diagnosis, diabetes mellitus (DM), and prostate disease. Predisposing factors include urinary catheter use, prior hospitalization, residing in a nursing home, and prior antibiotic treatment [8]. 

Measuring and comparing the AMR rates of hospital- and community-acquired UTIs is important because, while healthcare facilities experience the effects of AMR, the largest use of antimicrobials is in the community [9]. Although numerous studies have established the overall sensitivity and resistance spectrum for uropathogens [10, 11], only a few studies have considered whether the strains were isolated from hospital-acquired or community-acquired UTIs, a distinction that may affect the course of antibiotic therapy [12]. Since experimental antibiotic therapy for UTIs must be based on epidemiology and the uropathogen’s resistance pattern, this study is essential in terms of providing information about routine surveillance to reduce the therapy failure rate [13, 14]. 

Few studies were published in Egypt about the role of demographic and host-related factors associated with resistant urinary tract pathogens. However, both studies attributed AMR to *Enterococcus faecalis* among Egyptian patients with UTIs without studying the independent predictors of AMR [15, 16]. To the best of our knowledge, no previous studies monitoring the problem of AMR in hospital settings in Alexandria in the last decade were conducted. Our study fills the knowledge gap regarding AMR risk factors in the urology departments of our hospitals..

Our objective is to identify AMR rates in patients with UTIs and determine the factors associated with AMR among UTI-causing pathogens. This will improve strategies for AMR control and the rational use of antimicrobial drugs in UTIs.

## Materials and methods

### Study design, population, and sampling

We conducted an observational, retrospective, record-based study in the Urology Department of Alexandria University Hospital, one of the largest university hospitals in Egypt, which serves people who live in Alexandria and the North Delta.

In the extant literature, AMR prevalence in patients with UTIs varies between 40% and 69% [17, 18]. We hypothesized a 50% AMR prevalence in our patients. A minimum sample size of 371 patients achieves 80% power for estimating the expected proportion, with a maximum error estimate of ± 5% at a 95% confidence level and a 0.05 significance level. We recruited all adult patients—male or female—admitted to the Urology Department from October 2018 to October 2020. All patient details derived from the medical records were confidential. The study population included both community- and hospital-acquired UTIs.

### **Data collection**

Clinical specimens collected from urology patients were cultured, and the cultures were identified in the Department of Microbiology. We reviewed the patients’ medical records and extracted the following data from each record: demographic data (age, sex, and residence), admission diagnosis, chronic disease history, abnormal urinary tract structure, duration of hospital stay in days, urinary catheter insertion, catheter use duration in days, history of the use of antibiotics in the previous three months, Current urinary tract infection, History of previous urinary tract infection in the last year(if Yes: number of attacks, and the period between two attacks), history of urinary operation and the type of operation. If UTI was documented: we abstracted the following data: urine culture:(not done, done), use of antibiotics, results of urine cultures, number of pathogens, type of organism, and sensitivity of the organism. In the study, every urine culture was included once. If the affected person had more than one urine culture, the last result with the least missing clinical records has been chosen.

Urinary tract infection was defined by CDC as: patient clinically diagnosed by an attending physician, increased pus count in urine analysis and positive urine culture test. Clinical diagnosis depends on the following symptoms: dysuria, frequency, urinary incontinence, hematuria, suprapubic pain, offensive or turbid urine, changed or new vaginal discharge. Clinically associated symptoms may include; fever, chills, lower back ache or side back pain, nausea or vomiting [19]. Microbiologically urinary tract infection was defined as presence of greater than or equal to 10^5^ microorganisms CFU / 1 ml of urine with no more than two types of microorganisms or greater than or equal to 10^3^ according to the type of isolated micro-organisms and clinical situation of the patient [20–22]. 

We defined Community-acquired UTI as an infection of the urinary tract that occurs in the community or within less than 48 h of hospital admission and was not incubating at the time of hospital admission [23]. Hospital-acquired UTI was defined as patients free from UTI and the length of stay should be more than 48 h before symptoms of UTI appear to be sure that infection was acquired after admission to the urology department [24]. The 48-hour cut-off was due to the average time required by bacteria to develop in a human from initial infection to detection by a positive diagnostic test [25]. Urine samples collected were mid-stream urine and catheterized urine mainly.

Other operational definition of study variables is available in the supplementary file 1.

### The routine work for urine culture in the study hospital includes the following steps

Urine (clean-catch midstream) was collected in a sterile container, after thorough cleaning of the perineum and genitalia with soap and water several times (at least 3 times). The specimen was appropriately identified with the patient’s name and identification number, as well as other additional details such as the patient’s age. Other samples were also obtained as urine samples from the catheter.

The specimen was immediately transferred to the laboratory and refrigerated at 4 °C if a delay of more than two hours was anticipated. All samples were subjected to screening test using wet mount microscopic examination for estimation of polymorphonuclear leukocytes count, also dipstick test was used to screen for diagnosis of infection. All samples were inoculated on Blood agar with a Calibrated Loop (1 µl), MacConkey’s agar and Sabouraud dextrose agar aerobically at 35° C for 48 h.

Reading the Culture Results:

1. Plates were observed after 24 h of incubation for growth and the number of colonies were counted on blood agar.
2. Isolates were Identified, and sensitivities were performed as determined by CLSI guidelines [20, 21].

Urinary tract infection was diagnosed microbiologically:

Number of colonies × 1000 = cfu/ml when using calibrated loop 1 µl.

Alexandria University Diagnostic Medical Microbiology Lab is an ISO-accredited lab for 10 years (ISO 15,189 International Standard for Medical Laboratories). We undergo CAP competency testing. Panels included in accreditation are automated blood culture and urine culture. All interpretive criteria, specifically antibiotic sensitivity testing, are updated yearly as per the newly published CLSI guidelines. All isolated organisms diagnosed as pathogens are identified using the standard routine methods of identification by gram stain and biochemical reactions. In limited situations, vitek-2 compact system Biomerieux (available also in the lab) was used for identification when biochemical reactions were not conclusive because of the high cost of automated system consumables [21, 22]. 

Antibiotic sensitivity testing was performed routinely using the standard disc diffusion method and breakpoints for the results were interpreted according to the CLSI guidelines of each year. MIC was also performed by the broth microdilution method when colistin or vancomycin results were needed as recommended by the CLSI guidelines [26]. Automated MIC results from VITEK-2 system were also included in our results when performed in a selected number of our cases. We calculated the percentage of drug resistance as the number of drug resistant organisms in community or hospital acquired UTI (n) divided by number of organisms isolated in community or hospital acquired UTI (N) (DR = n/N x 100). Definitions of multidrug resistant isolates was considered as organism being non-susceptible to at least one agent in three or more antimicrobial categories, while that of extensively drug resistant was marked by their sensitivity to one or two classes of antibiotics only and pan drug resistance as resistant to all antibiotic classes suggested for therapy as proposed by European Centre for Disease Prevention and Control (ECDC) and the Centers for Disease Control and Prevention (CDC).

#### Statistical analysis

Qualitative variables were presented as percentages, and quantitative data as mean and standard deviation or median and interquartile range (IQR) according to the test of normality by Kolmogorov- Smirnov test. We assessed the association between qualitative variables and the outcome variable by Chi-square test. Fisher exact or Monte Carlo test adjustment was selected when 20% of cells or more have an expected value less than 5. Quantitative variables were compared by a one-way ANOVA test. Pairwise comparisons were conducted using the Post Hoc Games Howell test for age and duration of stay in the hospital and using the Post Hoc Gabriel test for the duration of use of the catheter. We calculated the cumulative incidence of UTI as the number of patients who developed UTI during their hospital stay within the study period divided by the total number of patients admitted during the study period [27]. 

Bivariate analysis was performed to detect which risk factors were associated significantly with antimicrobial resistance. We considered the following predictors:(sex, age, diagnosis, chronic disease, duration of stay in hospital, insertion of catheter, abnormal structure, previous use of antibiotics last three months, recurrent urinary tract infection, and type of operation). We included the variables in the final model after conducting bivariate analysis by Chi-square test. We conducted a multivariable logistic regression model using a stepwise backward method with a likelihood ratio test to assess the contribution of the previously mentioned predictors with respective Odds Ratio and 95% confidence Interval [28]. 

Model cross-validation was performed by randomly splitting the sample into development and test sets (ratio 3:1). By calculating the area under the receiver operating characteristics curve (AUROC) on the test set and the accuracy of the model’s predicted probability, the prognostic capacity of the model was assessed ^(51)^. We conducted Classification and Regression Tree Analysis (CART) for predicting drug resistance among patients with UTI. Cross-validation for assessing CART Model discrimination displayed by Receiver Operating Characteristic (ROC) curve. Heatmap briefly explained the sensitivity pattern of organisms to antibiotics. We used colors to represent sensitivity. All statistical analysis was two-sided, judged at 0.05 significance level and was performed using IBM SPSS statistics program version 28 and R software [29, 30]. 

#### Ethical consideration

We ensured anonymous data collection for keeping patients’ confidentiality. Medical Research Institute, Alexandria university Ethical Committee approved the research protocol.

## Results

**Fig. 1:**
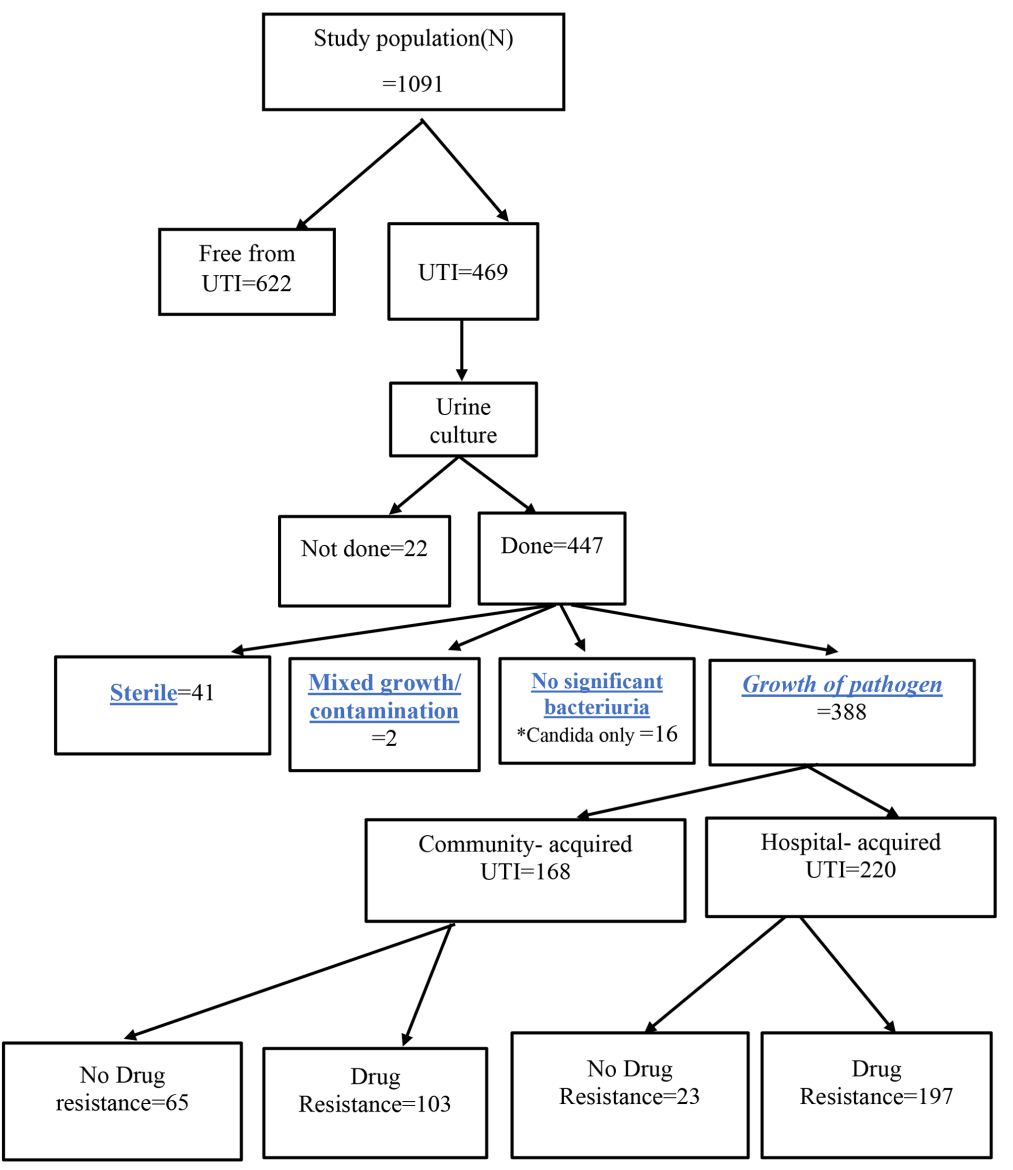
Algorithm showing results of drug resistance for patients with UTI admitted to urology department at Alexandria University Hospital. *Candida only: Cultures contain candida only not with other pathogens

The total number of patients recruited in the study was 1091, among which UTI was diagnosed in 469 patients (42.9%). Urine culture was performed for 447 (95.3%). Out of the 469 patients with UTI, pathogens were detected in 404 (90.4%) cases. Among 388 UTI patients with positive bacteria, 168 patients were diagnosed with community-acquired UTIs and 220 patients as hospital -acquired UTIs (Fig. 1).

Community-acquired UTI was diagnosed in 18.9% (206/1091) of patients admitted within the study period. During the patients’ hospitalization, the cumulative incidence of hospital-acquired UTIs was 29.7% (263/885). MDR was found in 67.7% (149/220) of patients with hospital-acquired UTI and in 49.4% (83/168) of cases with community-acquired UTI. Extensively-drug resistance (XDR) was identified in) 17.7% (39/220) of cases with hospital-acquired UTI and in 10.7% (18/186) of cases with community-acquired UTI. Pan-drug Resistance (PDR) was diagnosed in 4.1% (9/2202) of cases with hospital-acquired UTI and in 1.2% (2/168) of cases with community-acquired UTI.

There was an overall statistical difference in mean age between the three groups with mean age of 44.55 ± 21.07 in control group, was significantly higher than mean age of 39.3 ± 22.5 in community acquired group, while no statistical difference existed between both groups and hospital acquired group (40.5 ± 24.6 years).

(*P* = 0.003). Diagnosis of UTI was more frequent in female than male patients for both community and hospital acquired UTI respectively (88/118, 129/134, *p* < 0.001). The presence of stones of distinct types represents the commonest cause of admission in the control group (patients without UTI) (41.3%), community-acquired UTI (54.9%), and hospital-acquired UTI (40.7%). A highly statistically significant difference existed in the mean duration of hospital stays and is higher in hospital-acquired UTI (13.24 ± 6.56) than community-acquired UTI (11.58 ± 4.29), and control group (10.3 ± 3.2). (*P* < 0.001). Table 1, supplemental Fig. 1.

**Table 1:** Demographic and clinical characteristics of the study population admitted to urology department at Alexandria University Hospital from October2018 to October2020 (*N* = 1091)

	Patients without UTI (control) (*N* = 622) N (%)	Community- acquired UTI (*N* = 206) N (%)	Hospital -acquired UTI (*N* = 263) N (%)	Sig.
Age(years) Mean ± SD	44.55 ± 21.07^a^	39.3 ± 22.5^b^	40.5 ± 24.6^a,b^	***P*** = 0.003*
Gender: Male/Female	461/161 ^a^	88/118 ^a^	129/134 ^b^	***P*** < 0.001*
Diagnosis on admission(%yes): Stones Tumors Others	257(41.3%) ^a^ 181(29.1%) ^a^ 184(29.6%)	113(54.9%) ^a, b^ 42(20.4%)^a, b^ 51(24.7%)	107(40.7%) ^a^ 75(28.5%) ^b^ 81(30.8%)	0.01* 0.030* 0.224
Abnormal structure of urinary tract: Yes No	49(7.9%) ^a^ 573(92.1%) ^a^	30(14.6%) ^b^ 176(85.4%) ^b^	58(22.1%) ^b^ 205(77.9%) ^b^	***P*** < 0.001*
Use of urinary catheter: Yes No	77(12.4%) ^a^ 545(87.6%) ^a^	82(39.8%) ^a^ 124(60.2%) ^a^	135(51.3%) ^b^ 128(48.7%) ^b^	***P*** < 0.001*
Duration of use of catheter(days) Mean ± SD	10.75 ± 3.27^a^	11.86 ± 4.24^a^	12.90 ± 3.8^b^	p.001*
Recurrent UTI: Yes No	0(0%) ^a^ 622(100%) ^a^	104(50.5%) ^a^ 102(49.5%) ^a^	148(56.3%) ^b^ 115(43.7%) ^b^	***P*** < 0.001*
Duration of stay in hospital(days): Mean ± SD	10.3 ± 3.2 ^a^	11.58 ± 4.29 ^b^	13.24 ± 6.56 ^c^	***P*** < 0.001*
Previous use of antibiotics within last three months Yes No	111(17.8%) ^a^ 511(82.2%) ^a^	120(58.3%) ^b^ 86(41.7%) ^b^	186(70.7%) ^c^ 77(29.3%) ^c^	***P*** < 0.001*
Urinary operation: Yes No	590(94.9%) 32(5.1%)	187(90.8%) 19(9.2%)	249(94.7%) 14(5.3%)	0.089

Diagnosis: (i) Stones in kidney, urethra, and bladder. (ii) Tumors (bladder cancer, prostate cancer, renal tumor) (iii) Others (renal failure, dysuria, erectile dysfunction, hypospadias, ureteral stricture, urinary incontinence, hematuria, undescended testis, prostate hyperplasia) Diabetes mellitus was present as comorbidity in control group was 50(8%), in Community- acquired UTI group was 28(13.6%), and in hospital-acquired UTI group was 67(25.5%), there was statistically significant difference between groups (*P* < 0.001). ^a, b, c^:Different superscript denote significant pairwise comparison with adjusted significance

### Microbiological etiology of UTI

Each patient had one culture with a total positive culture of 404 and total organisms of 422. Gram-negative bacteria represented 372(88.2%) out of all organisms, Gram-positive bacteria represented 25(5.6%), and Candida represented 25(5.6%) of the total number of organisms. There is no urogenital flora. The most common organisms were E. coli 224(53.3%), followed by Klebsiella pneumonia 89(21.2%), Proteus mirabilis 22(5.2%), and Pseudomonas aeruginosa 17(4%). Table 2.

**Table 2:** Distribution of the isolated bacteria from patients with hospital acquired UTI and community acquired UTI

Bacteria	Hospital-acquired UTI *N* = 251 N (%)	Community- acquired UTI *N* = 171 N (%)	Total *N* = 422 N (%)
*E. coli*	132(52.6%)	92(53.8%)	224(53.1%)
*Klebsiella pneumoniae*	57(22.7%)	32(18.7%)	89(21.1%)
*Candida*	19(7.6%)	6(3.5%)	25(5.9%)
*Proteus mirabilis*	8(3.2%)	14(8.1%)	22(5.2%)
*Pseudomonas aeruginosa*	11(4.4%)	6(3.5%)	17(4%)
*Acinetobacter baumannii*	10(3.9%)	5(3%)	15(3.6%)
Others	14(5.6%)	16(9.4%)	30(7.1%)

Others including *Burkholderia cepacia, Providencia stuartii, Enterococcus faecalis, Staphylococcus aureus, Coagulase negative staphylococci, Streptococcus pyogenes* species

Cultures that had one pathogen represented 386(95.5%) and two pathogens represented 18 (4.5%). We detected 125 (30.9%) cultures with greater than or equal to 103 and less than or equal to 105 microorganisms and 279 (69.1%) cultures with greater than or equal to 105 microorganisms / 1 cm3 of urine. ESBL - E. coli were 63(28.1%) isolates and 161(71.9%) non-ESBL isolates. ESBL- Klebsiella were 9(10.1%) isolates, and 80(89.9%) non-ESBL isolates. Organisms isolated from hospital-acquired UTI were 59.5%, while 40.5% of organisms isolated from the community- acquired UTI.

### Percentage of drug resistance (DR) presented for each isolated organism

**Fig. 2:**
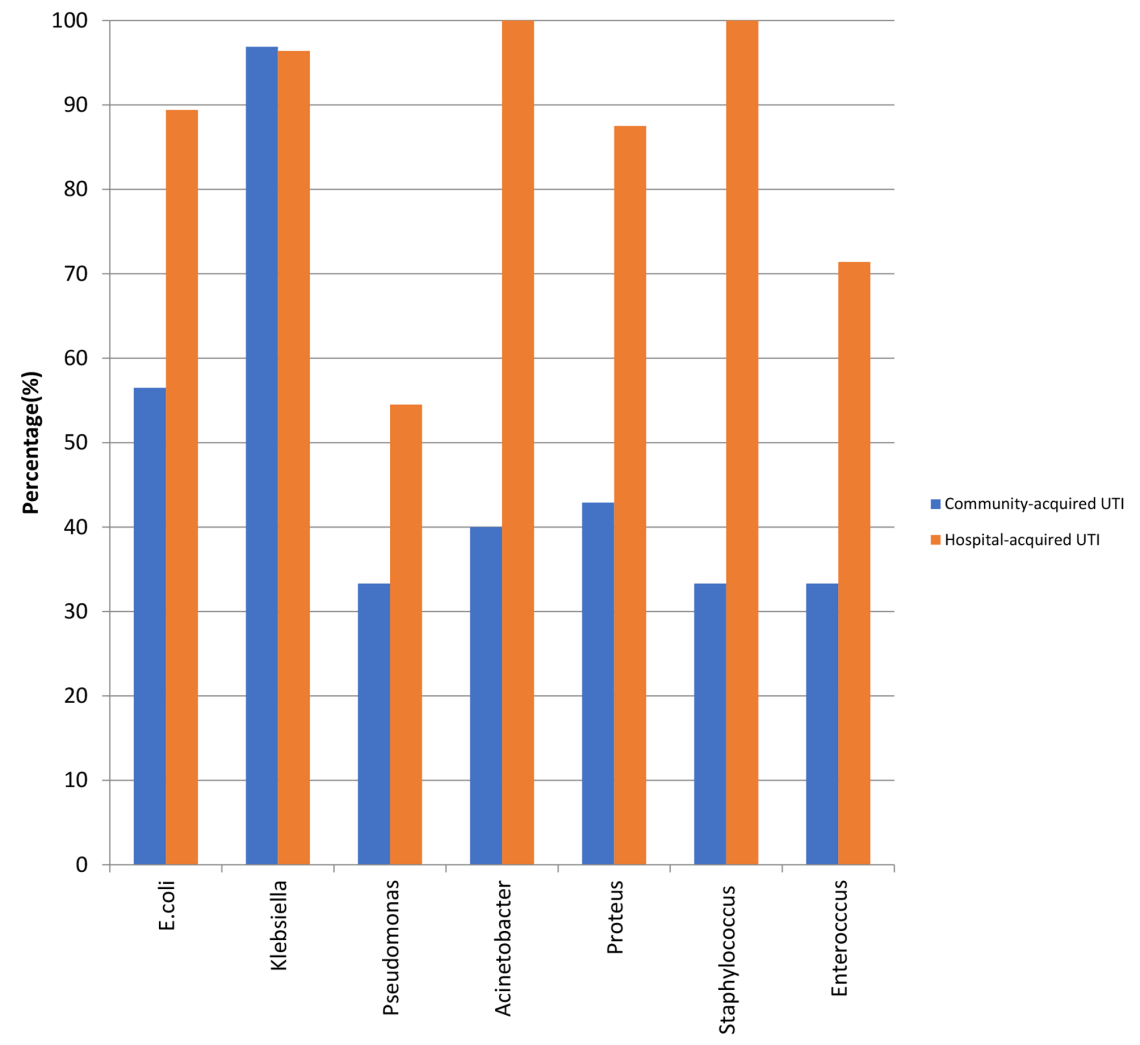
Percentage of drug resistance (DR) presented for isolated organisms from community and hospital- acquired UTI

The percentage of drug resistant Klebsiella, E. coli, Proteus, Acinetobacter, Pseudomonas, Staphylococcus, and Enterococcus species in community- acquired UTI was 96.9% (31/32), 56.5% (52/92), 42.9% (6/14), 40% (2/5), 33.3% (2/6), 33.3% (1/3), 33.3% (1/3) respectively. The percentage of drug-resistant of Acinetobacter, Staphylococcus, Klebsiella, E. coli, Proteus, Enterococcus, and Pseudomonas species in hospital-acquired UTI was 100% (10/10), 100% (6/6), 96.4% (55/57), 89.4% (118/132), 87.5% (7/8), 71.4% (5/7), 54.5% (6/11) respectively. Figure 2.

There was a statistically significant difference in the proportion of drug-resistant of E. coli, and Acinetobacter species between community and hospital-acquired UTI (*P* < 0.001, 0.022 respectively). The percentage of drug-resistant of E. coli, Pseudomonas, Acinetobacter, Proteus, Enterococcus, and Staphylococcus species was higher in hospital-acquired UTI than community-acquired UTI, while the percentage of drug-resistant of Klebsiella was higher in community-acquired UTI than hospital-acquired UTI.

## Antibiogram

**Fig. 3:**
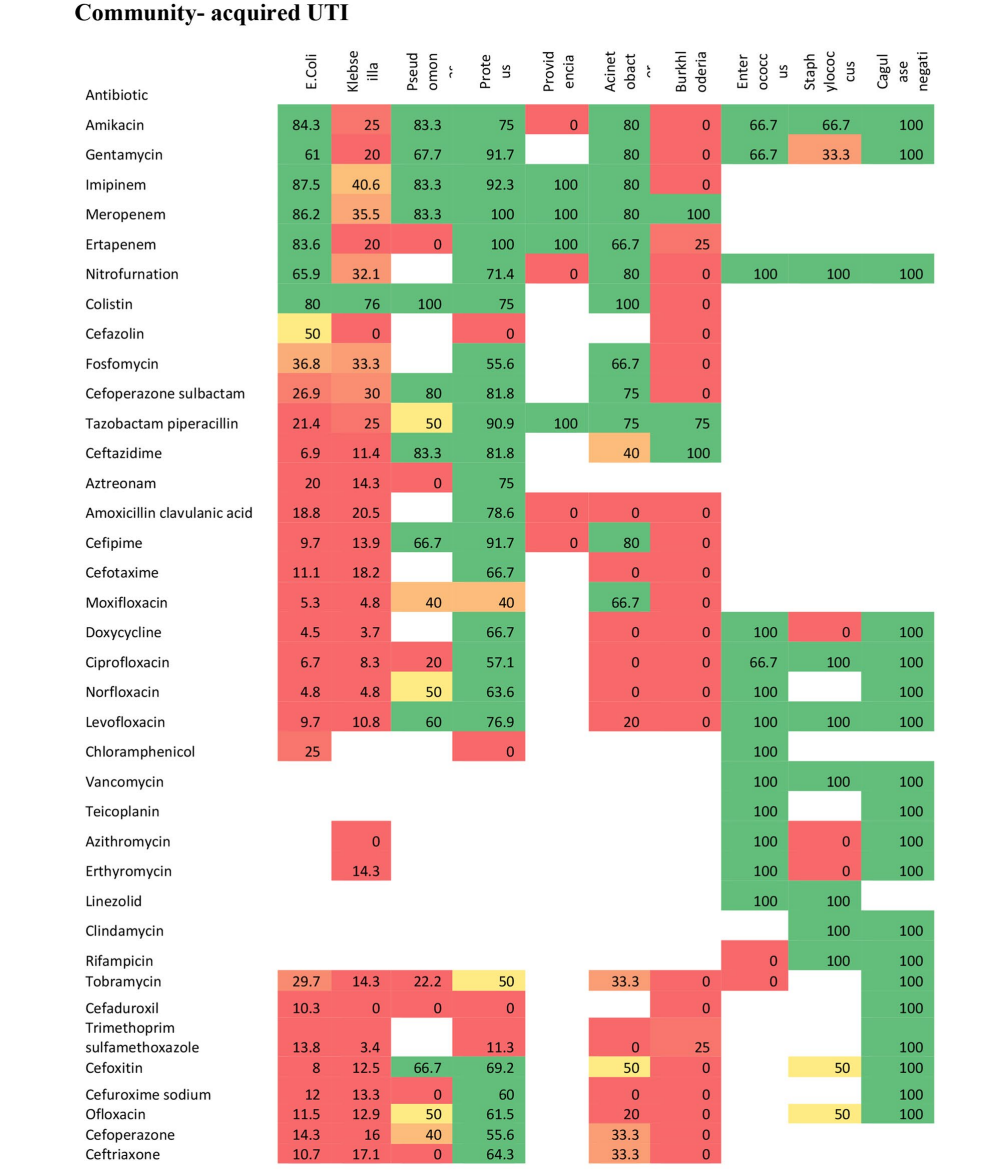
Heat map of sensitivity of organisms to different antibiotics in community -acquired UTI. Green color indicates high sensitivity (51-100%), Orange and yellow colors indicate moderate sensitivity (23-50%), Red color indicates low sensitivity (0-22%), empty cells for not assessed antibiotics

**Fig. 4:**
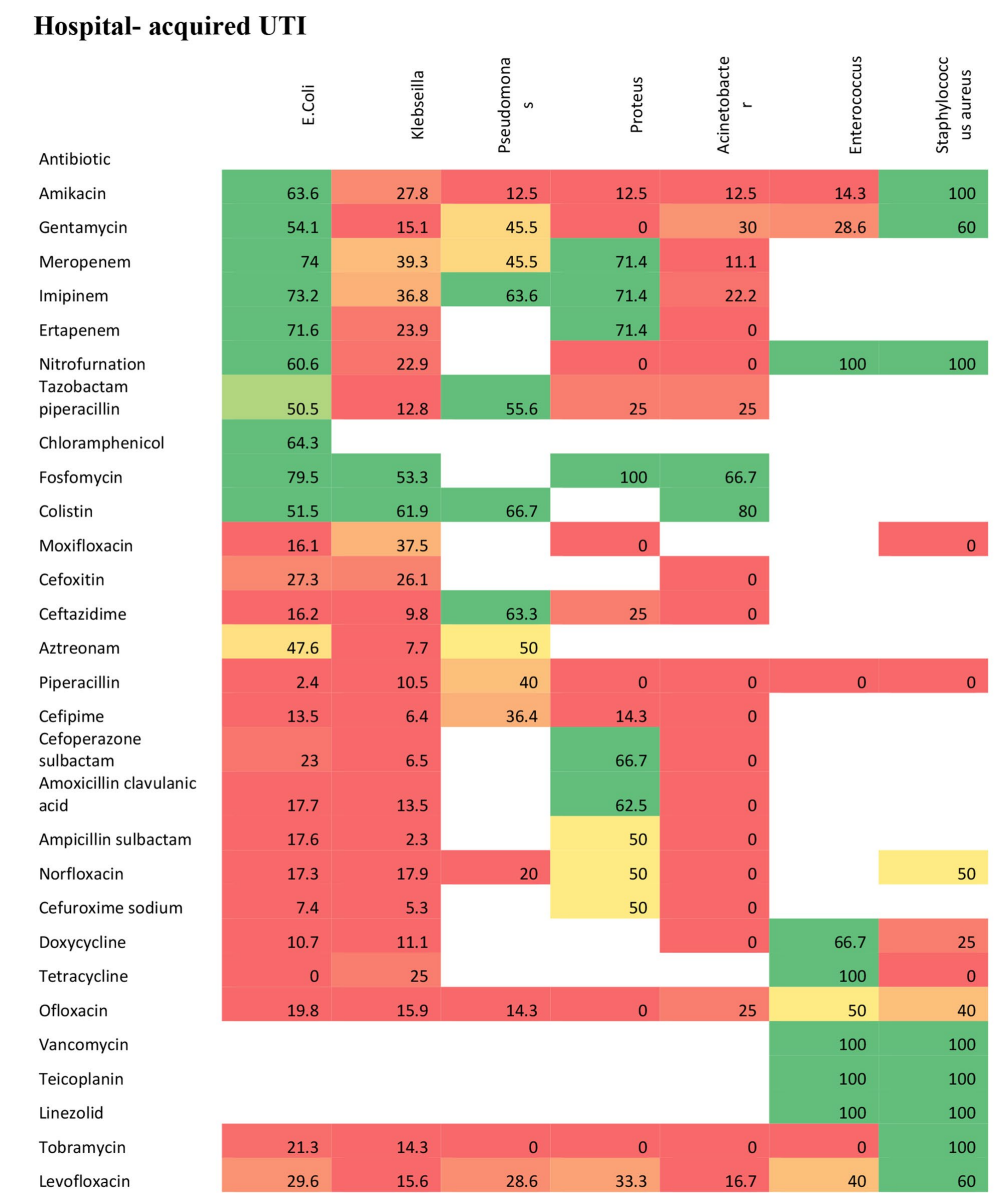
Heat map of sensitivity of organisms to different antibiotics in hospital- acquired UTI. Green color indicates high sensitivity (51-100%). Orange and yellow colors indicate moderate sensitivity (23-50%). Red color indicates low sensitivity (0-22%). Empty cells for not assessed antibiotics

We displayed an antibiogram per organism for community-acquired and hospital-acquired UTI. We illustrated antibiogram for E. coli and Klebsiella pneumoniae as the most common isolated organisms. Other organisms are illustrated in detail as shown in Figs. 3 and 4 and supplemental file 1.

### Antibiogram of E. Coli

In community acquired UTIs, the top five most susceptible antibiotics against E. coli were Imipenem, Meropenem, Amikacin, Ertapenem, and Colistin. In the hospital acquired UTIs, the top five most susceptible antibiotics against E. coli were Fosfomycin, Meropenem, Imipenem, Ertapenem, and Chloramphenicol.

### Antibiogram of Klebsiella pneumoniae

In community acquired UTIs, the top three most susceptible antibiotics against Klebsiella were Colistin, Imipenem, and Meropenem. In hospital acquired UTIs, the top two most susceptible antibiotics against Klebsiella were Colistin, and Fosfomycin.

### Bivariate and multivariable analysis for drug resistance

On bivariate analysis, Drug Resistance was significantly associated with the presence of recurrent UTI (OR = 6.7, *P* < 0.001), tumor disease (OR = 2, *P* = 0.021), stones (OR = 1.8, *P* = 0.021), hospital-acquired UTI (OR = 5.3, *P* < 0.001), diabetes mellitus (OR = 8.3, *P* < 0.001), and old age (OR = 2.7, *P* < 0.003). Drug Resistance was significantly associated with predisposing factors on bivariate analysis such as previous use of antibiotics within last three months (OR = 9.3, *P* < 0.001), long duration of stay in hospital ≥ 15 days (OR = 2.4, *P* = 0.020), and PCNL operation (OR = 1.9, *P* = 0.008). Statistically significant and clinically relevant variables from the bivariate analysis were included in the multivariable logistic regression to show the independent predictors of Drug Resistance.

The risk factors independently associated with Drug Resistance on multivariable logistic regression were patients with hospital-acquired UTI (AOR = 5.7, 95% CI 3.06–10.76, *p* < 0.001). Patients who had hospital-acquired UTI had 5.7 times more chance to develop drug resistance than patients who had community-acquired UTI. Patients who were older than or equal to 60 years had 2.9 times more chance to develop drug resistance than patients who were younger than 60 years (AOR = 2.9, 95% CI 1.27–6.72, *P* = 0.012). Patients with recurrent UTI had 2.6 times more chance to develop drug resistance than patients who had no recurrent UTI (AOR = 2.6, 95% CI 1.08–6.20, *P* = 0.033). Patients with DM as a comorbidity had 3.8 times more chance to develop drug resistance than patients who had no DM (AOR = 3.8, 95% CI 1.24–11.84, *P* = 0.019). Finally, patients who used antibiotics within the last three months had 5.2 times chance to develop drug resistance than patients who did not use antibiotics within the same period (AOR = 5.2, 95% CI 2.19–12.31, *P* < 0.001). Table 3.

**Table 3:** Bivariate and Multivariable logistic regression analysis for predicting the independent contribution of potential predictors of antimicrobial resistance among patients with UTIS

Variables	Drug Resistance					
	Yes n(%)	No n(%)	Unadjusted OR (95%CI)	Sig.	Adjusted OR (95%CI)	Sig.
Hospital acquired UTI(*N* = 220)	197(89.5%)	23(10.5%)	5.3 (3.2-9)	***P*** < 0.001*	5.7 (3.058–10.76)	***P*** < 0.001*
Female(*N* = 218)	173(79.4%)	45(20.6%)	1.3 (0.83–2.14)	0.236		
Age:≥60(*N* = 101)	89(88.1%)	12(11.9%)	2.7 (1.4–5.22)	0.003*	2.9 (1.266–6.720)	0.012*
Tumor:(*N* = 108)	92(85.2%)	16(14.8%)	2 (1.11–3.64)	0.021*		
Stones:(*N* = 164)	117(71.3%)	47(28.7%)	1.8 (1.09–2.82)	0.021*		
Diabetes mellitus:(*N* = 88)	84(95.5%)	4(4.5%)	8.3 (2.9–23.4)	***P*** < 0.001*	3.8 (1.243–11.837)	0.019*
Abnormal structure of urinary tract:(*N* = 81)	61(75.3%)	20(24.7%)	0.9 (0.5–1.56)	0.663		
Use of urinary catheter:(*N* = 190)	154(81.1%)	36(18.9%)	1.6 (0.96–2.5)	0.072		
Recurrent UTI: (*N* = 246)	220(89.4%)	26(10.6%)	6.7 (3.9-11.25)	***P*** < 0.001*	2.6 (1.083–6.202)	0.033*
Continuous stay in hospital:≥15 days(*N* = 73)	64(87.7%)	9(12.3%)	2.4 (1.15–5.1)	0.020*		
Previous use of antibiotics within last three months:(*N* = 284)	252(88.7%)	32(11.3%)	9.3 (5.5-15.87)	***P*** < 0.001*	5.2 (2.186–12.307)	***P*** < 0.001*
PCNL:(*N* = 121)	83(68.6%)	38(31.4%)	1.9 (1.19–3.18)	0.008*		

*Statistically significant variables by bivariate analysis were initially included in multivariate logistic regression using backward likelihood ratio method were stones, tumor, diabetes mellitus, recurrent UTI, duration of stay in hospital, previous use of antibiotics, old age, patients with hospital- acquired UTI, PCNL.

*44.8% of the variance of the drug resistance outcome is explained by significant independent variables included in the model (McFadden R^2^ = 0.448), Model Summary:X^2^ = 136.022, *P* < 0.001

Hosmer-Lemeshow X^2^ = 8.743, *P* = 0.189, PCNL: Percutaneous nephrolithotomy

### Classification and regression tree analysis (CART) for predicting drug resistance among patients with UTI

**Fig. 5:**
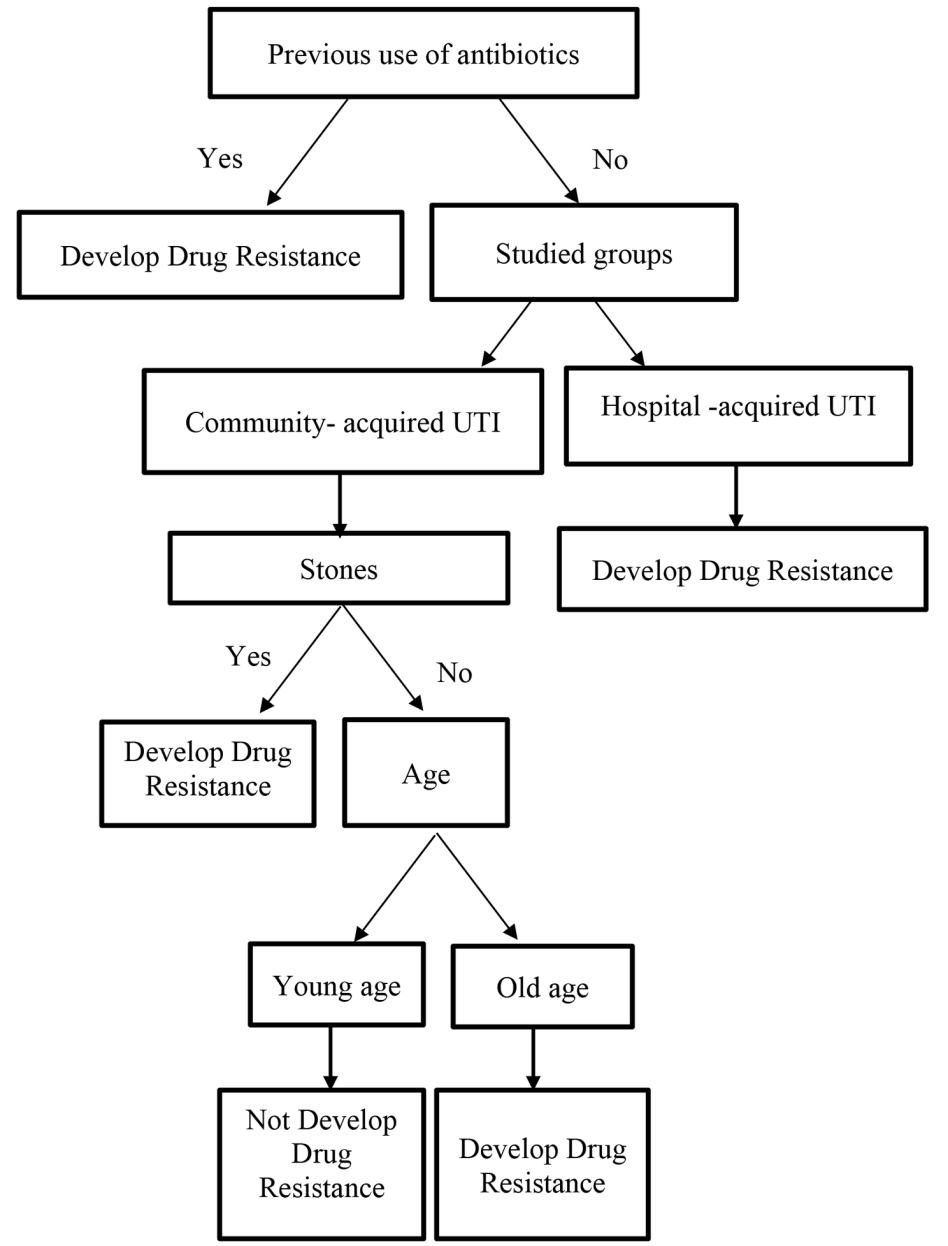
Classification and Regression Tree Analysis (CART) for predicting drug resistance among patients with UTI

Applying CART analysis, previous use of antibiotics within last three months is the most significant predictor for developing drug resistance, Patients who had a history of previous use of antibiotics within last three months were most probably to develop drug resistance. Patients without a history of use of antibiotics and with hospital-acquired UTI were more liable to develop drug resistance. Those patients with community-acquired UTI and who had a previous history of stones were also more liable to develop drug resistance. However, if negative, drug resistance was less likely to occur especially among early-age patients. Figure 5.

The logistic regression model as well as the CART model proved high discrimination and accuracy on the test set as demonstrated by AUC of 0.881, 0.854 respectively (Fig. 5, Supplemental Figs. 2 and 1).

## Discussion

Antimicrobial stewardship is more demanding in developing Countries in terms of the governance of the health sector [31, 32]. In the present study, we concluded that both the incidence of UTI and the proportion of drug resistance are strikingly high among hospitalized patients in the urology department. The cumulative incidence of hospital-acquired UTI among our study population is 29.7%. This proportion is higher than a study conducted in Portugal which reported a cumulative incidence of 4.6% (95% CI: 2.5–6.7) [33]. 

Community-acquired UTI was detected in 18.9% of the patients. This figure is similar to findings from other developing countries like Rwanda and India (19.3%, and 10.9% respectively) [11, 14]. Higher incidence proportions were reported in studies from Cameroon and Nigeria (59.8% and 39.7%. respectively) [34, 35]. The incidence difference might be attributable to various levels of infection control, variations in the methods and/or operational definition of positive UTI. In concordance with other studies, E. coli was the most prevalent organism (53.3%) that was detected from urine samples in both hospital and community-acquired UTIs [36–38]. Similarly, the second most common isolate in the present study was Klebsiella pneumoniae (21.2%) [12, 39, 40]. This is different from a prior research by Tessema et al., [41] that reveled the second most frequently isolated pathogen was Staphylococcus species. Previous studies in Iran, India, and Korea reported that the second most common bacterial isolate was Enterococcus faecalis [42–44]. The similarities and variations in the type and distribution of uropathogens might be due to a variety of environmental variables, host characteristics and laboratory methods, as well as hygienic standards, in each country [45–47]. 

It is important to note that E. coli and Klebsiella species together cause around three quarters of all cases of UTIs in our study population. Focusing on the best antibiotics selection which can successfully treat these two organisms can guide empirical treatment whenever needed. Our study displayed that both E. coli or Klebsiella species isolated from the community or hospital-acquired UTIs are mostly sensitive to Meropenem, Imipenem, Fosfomycin, Nitrofurantoin, and Colistin antibiotics.

In community-acquired UTI, our results indicated high sensitivity of E. coli to Imipenem, Meropenem, Ertapenem, Nitrofurantoin Gentamycin, and Amikacin. This was supported by similar studies that demonstrated the sensitivity of E. coli strains to Imipenem was 93%. High susceptibility to Fosfomycin, nitrofurantoin, and gentamicin was also observed (60%, 60%, and 78%, respectively) [42, 48]. We justified the findings by the nature of community acquired UTI with less exposure to antibiotics versus the virulent nature of organisms causing hospital acquired infections.

In hospital-acquired UTI, our findings displayed high sensitivity of E. coli to Fosfomycin, Imipenem, Nitrofurantoin and Gentamycin (79.5%,73.2%, 60.6%, and 54.1% respectively). This study displayed that Klebsiella in community-acquired UTI was reasonably sensitive to colistin (76%), imipenem (40.6%), and meropenem (35.5%). High resistance was reported to other antibiotics; Ampicillin, Trimethoprim sulfamethoxazole, and Nitrofurantoin. The sensitivity of Klebsiella to Fosfomycin in hospital-acquired UTIs was (53.3%). Comparable findings were detected in a systematic review that reported 77% susceptibility of Klebsiella to Fosfomycin [49]. While this study concluded high resistance to other antibiotics, the same review displayed that Klebsiella was sensitive to Ofloxacin (73%); Ciprofloxacin (74%); Gentamicin (69%); Tobramycin (70%); Amikacin (97%) [49]. 

Our results reported that Pseudomonas in community-acquired UTI was extremely sensitive to Colistin 100%, Imipenem 83.3%, Meropenem 83.3%, and Ceftazidime 83.3%. Comparable results for sensitivity to Colistin, Amikacin, Gentamycin, and Cefepime were reported in a previous study conducted in the United States [50]. We also concluded that Proteus in community-acquired UTI was highly resistant to ampicillin (100%). A research was conducted in Cameron and revealed that proteus was sensitive to Fosfomycin 55% but resistant to Fosfomycin [34]. Regional disparities in strain prevalence, as well as different strategies of using antibiotics, might explain the observed variances in resistance rates [51]. 

Regarding risk factors related to drug resistance, our findings indicated that antibiotic resistance proportions are greater in hospital-acquired UTIs than in community-acquired UTIs. The only exception is for Klebsiella in which drug resistance is strikingly high (96%) in both hospital-acquired, and community-acquired UTIs. This finding is consistent with other studies [7, 52]. The higher antibiotic resistance in hospital-acquired UTIs could be related to many factors such as the use of invasive medical procedures, extensive prescription of broad-spectrum antibiotics, and inadequate hospital infection control methods [53]. For other risk factors related to antimicrobial resistance, we found that malignancy, renal stones, previous use of antibiotics within the last three months, recurrent UTI, diabetes mellitus, old age, and prolonged stay in hospital ≥ 15 days are all associated with higher proportions of resistance. Using multivariable logistic regression to adjust for confounding effect, independent predictors for drug resistance include hospital-acquired UTI, old age, recurrent UTI, DM, and previous use of antibiotics within the last three months. In concordance to our results, Khawcharoenporn et al. [54] displayed that age, male gender, DM, obstructive uropathy, recurrent UTI, and prior use of any antibiotics within the preceding 3 months were all connected to MDR UTIs. Previous use of antibiotics associated with antimicrobial resistance was the most common reported risk factor in previous research [28, 55, 56]. Hospital-acquired UTI as risk factor for antimicrobial resistance was also detected in other studies [8, 54]. 

Previous research displayed old age [54, 55]. The link between increasing age and greater resistance is unsurprising, given that aging’s physiological changes and increased comorbidities predispose to a higher chance of infection, resulting in more visits to healthcare facilities and therefore more antibiotic exposure [57]. In concordance to previous studies, we reported DM as a risk factor for antimicrobial resistance [8, 54, 55]. In our study, we also found recurrent UTI as a risk factor for antimicrobial resistance which supports findings in previous research [8, 58, 59]. 

Among the strengths of our study is the large sample size. Also, the generalizability of our findings to other healthcare facilities in Alexandria, Egypt is assumed because the study hospital is the largest tertiary healthcare facility in Alexandria and receives referrals from other hospitals in the city. Antibiotic stewardship program in our health care facility was initiated in the last two years aiming to limit antibiotic abuse and to designate responsible personnel for prescribing valuable antibiotics and save these antimicrobials for significant infections and deserving situations.

One potential limitation is that our study is retrospective record-based research, with the possibility of information bias. However, the key variables in our study are objective and appropriately documented in medical records.

### Conclusion and clinical implications

Based on the results of the present study, the most common isolated organisms from hospitalized patients in the urology department include E. coli, Klebsiella pneumonia, Proteus mirabilis, and Pseudomonas aeruginosa.

The best drug choices include Imipenem, Meropenem, Fosfomycin, Nitrofurantoin, and Colistin.

Antibiotic resistance cumulative incidence was greater in hospital-acquired UTIs than in community-acquired UTI.

Independent predictors of antimicrobial resistance were a history of previous use of antibiotics within last three months, the occurrence of hospital-acquired UTI, DM, old age patient, and recurrent UTI. This will guide doctors in recognizing patients with a high risk of developing antimicrobial resistance.

Considering this worrying problem of antibiotic resistance and the emergence of multidrug-resistant bacterial strains which hinder the global control of infectious diseases, further research is required for promising new biological compounds against multidrug-resistant organisms that are innovative compared to traditional antibiotics. Empirical therapy for the treatment of UTI should be tailored to the susceptibility of potential pathogens to ensure optimal treatment. Rational use of antibiotics is essential to prevent further increase of AMR.

### Electronic supplementary material

Below is the link to the electronic supplementary material.

Supplementary Material 1

Supplementary Material 2

## Data Availability

All data are available upon request by emailing the corresponding author.

## Declarations

### Human approval

We ensured anonymous data collection for keeping patients’ confidentiality. MRI Ethical Committee approved the research protocol. All methods were carried out in accordance with relevant guidelines and regulations.

## Abbreviations

AMR: Antimicrobial resistance
CART: Classification and Regression Tree Analysis
E. coli: Escherichia coli
ESBL: Extended Spectrum Beta-Lactamase
MDRO: Multi-Drug Resistant Organisms
MDR: Multi-Drug Resistant
PCNL: Percutaneous Nephrolithotomy
PDR: Pan Drug Resistant
ROC: Receiver Operating Characteristic
UTI: Urinary Tract Infection
XDR: extensively Drug Resistant
