# Supplementary material for: Trends and predictors of antimicrobial resistance among patients with urinary tract infections at a tertiary hospital facility in Alexandria, Egypt: a retrospective record-based classification and regression tree analysis
Source: BMC Infect Dis. 2024 Feb 22;24:246. doi: 10.1186/s12879-024-09086-6 (PMC10885625; doi:10.1186/s12879-024-09086-6)
Supplement: Supplementary file 1 — Supplementary Material 1 [file 12879_2024_9086_MOESM1_ESM.docx]

**Operational definition of variables**

**Recurrent urinary tract infection**

Refers to two infections in six months or three infections in one year. ^(3)^

**Empirical treatment**

Treatment given without knowledge about the cause of the disorder and based on experience as a substitute than common sense. Sometimes urgency dictates empirical treatment, as when a risky contamination by an unknown organism is treated with a broad -spectrum antibiotic while the results of bacterial culture and other investigations are awaited. ^(2)^

**Antibiotic resistance**

A scenario that happens when the efficacy of a medication which is used in the treatment of bacterial infections is compromised. ^(1,4)^

**Intermediate**

It refers to isolates that are "moderately sensitive" to antibiotics. The intermediate category acts as a buffer zone between the susceptible and resistant categories. ^(5)^

**Antibiotic susceptibility tests**

Used to assess how the pathogen will respond to antibiotic treatment. Used in the management of diseases by providers. "Reported as a "MIC" or "minimum inhibitory concentration. ^(6)^

The antibiogram was performed by disc diffusion Kirby-Bauer method.

Catheter-associated urinary tract infection

A urinary tract infection (also called “UTI”) is an infection in the urinary system, which includes the bladder (which stores the urine) and the kidneys (which filter the blood to make urine). Germs (for example, bacteria or yeasts) do not normally live in these areas; but if germs are introduced, an infection can occur. If you have a urinary catheter, germs can travel along the catheter and cause an infection in your bladder or your kidney; in that case it is called a catheter-associated urinary tract infection (or “CA-UTI”).^(7)^

**References:**

1-Clements, A. C., Magalhães, R. J., Tatem, A. J., Paterson, D. L., & Riley, T. V. (2010). Clostridium difficile PCR ribotype 027: assessing the risks of further worldwide spread. *The Lancet Infectious Diseases*, *10*(6), 395–404. <https://doi.org/10.1016/S1473-3099(10)70080-3>

2-Free Dictionary. (2004). Empirical treatment. Definition of empirical treatment by Medical dictionary.

https://medical dictionary.thefreedictionary.com/empirical+treatment

3-Glover, M., Moreira, C. G., Sperandio, V., & Zimmern, P. (2014). Recurrent urinary tract infections in healthy and nonpregnant women. *Urological Science*, *25*(1), 1–8. <https://doi.org/10.1016/j.urols.2013.11.007>

4-Smith, R. A., M’ikanatha, N. M., & Read, A. F. (2015). Antibiotic Resistance: A Primer and Call to Action. Health Communication, 30(3), 309–314. https://doi.org/10.1080/10410236.2014.943634

5-Tankeshwar, A. (2022). What does Susceptible, Intermediate, and Resistant Mean?. https://microbeonline.com/interpretation-susceptibility-testing-susceptible-intermediate-resistant-mean/

6-Your, R., Credits, C. E., & Broker, C. E. (2014). Pharmacologic Resistant Microorganisms: A Modern Day Plague.

7- <https://www.cdc.gov/hai/pdfs/uti/ca-uti_tagged.pdf>

The error bar chart (Figure 1) shows that the mean of duration of stay in hospital is higher in hospital acquired UTI (13.24±6.56) than community acquired UTI (11.58±4.29), and control group (10.3±3.2).


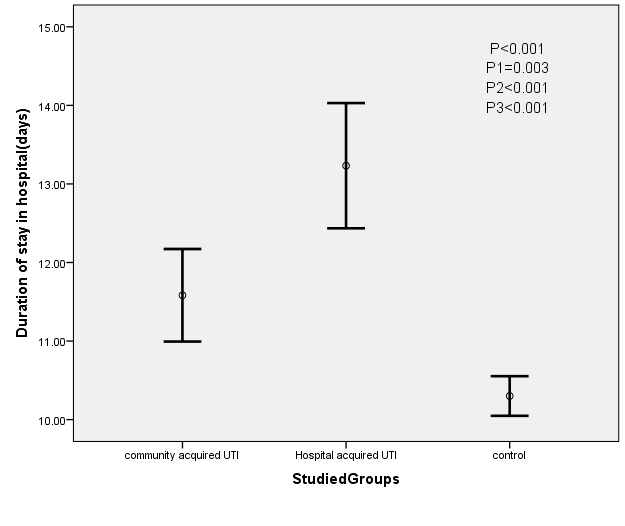


**Supplemental Figure (1):** Error bar of mean duration of stay in hospital among patients with hospital acquired, community acquired UTI, and the control group.

P: P value for comparing between the studied groups.

P1: P value for comparing between community acquired UTI and hospital acquired UTI.

P2: P value for comparing between community acquired UTI and control.

P3: P value for comparing between hospital acquired UTI and control.

**Antibiogram:**

**Antibiogram of Pseudomonas aeruginosa**

In community acquired UTI Pseudomonas displayed high sensitivity to Colistin 100%, Ceftazidime 83.3%, Amikacin 83.3%, Imipenem 83.3%, Meropenem 83.3%, Cefoperazone sulbactam 80%, , Ceftriaxone 75%, Gentamycin 66.7%, Cefoxitin 66.7%, Cefepime 66.7%, Levofloxacin 60%, but showed high resistance to Ampicillin 100%, Piperacillin100%, Cefaclor 100%, and Cefuroxime sodium 100%.

In hospital acquired UTI Pseudomonas was overly sensitive to Colistin 66.7%, Imipenem 63.3%, Ceftazidime 63.3%, and Tazobactam piperacillin 55.6%, while displayed maximum resistance to Tobramycin 100%, Ampicillin 100%, Norfloxacin 80%, Amikacin 75%, Ciprofloxacin 75%, Ofloxacin 71.4%, and Levofloxacin 71.4%.

**Antibiogram of Proteus mirabilis**

In community acquired UTI, Proteus displayed high sensitivity to Meropenem 100%, Ertapenem 100%, Imipenem 92.3%, Gentamycin 91.7% Cefepime 91.7%, Tazobactam piperacillin 90.9%, Ceftazidime 81.8%, Cefoperazone sulbactam 81.8%, Amoxicillin clavulanic acid 78.6%, Levofloxacin 76.9%,Amikacin 75%, Aztreonam 75% , Colistin 75%, Nitrofurantoin 71.4% Cefoxitin 69.2%, Doxycycline 66.7%, Cefotaxime 66.7%, Doxycycline 66.7%, Ceftriaxone 64.3%, Norfloxacin 63.6%, Ofloxacin 61.5%, Cefuroxime sodium 60%, Cefoperazone 55.6%, Fosfomycin 55.6%, and Ciprofloxacin 57.1%, but was highly resistant to Rifampicin 100%, Cephradine 100%, Cefaclor 100%, Ampicillin 100%, Piperacillin 100%, Tetracycline 66.7%, and Cefadroxil 66.7%.

In hospital acquired UTI, Proteus was highly sensitive to Fosfomycin 100%, Imipenem 71.4%, Meropenem 71.4%, Ertapenem 71.4%, Cefoperazone sulbactam 66.7%, and Amoxicillin clavulanic acid 62.5%, while exhibited maximum resistance to Ampicillin 100%, Cephalothin 100%, Cefoperazone 100%, Moxifloxacin 100%, Ceftriaxone 87.5%, Amikacin 86.5%, Gentamycin 86.5%, Cefotaxime 85.7%, Cefepime 85.7%, Ofloxacin 85.7%, Nitrofurantoin 83.5%, Trimethoprim sulfamethoxazole 75%, Ceftazidime 75%, and Levofloxacin 66.7%.

-
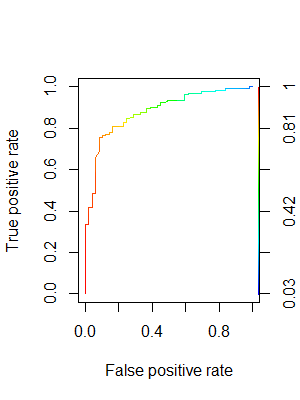


**Supplemental figure (2):** Cross validation for assessing logistic regression model’s discrimination displayed by Receiver Operating Characteristic (ROC) curve, AUC= 0.881.


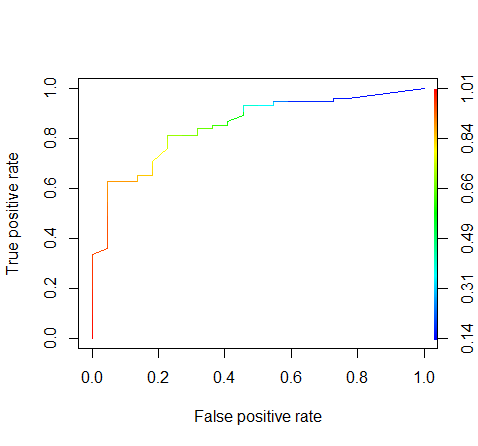


**Supplemental figure (3):** Cross validation for assessing logistic regression CART’s discrimination displayed by Receiver Operating Characteristic (ROC) curve, AUC= 0.854.
