# Supplementary material for: Trends and predictors of antimicrobial resistance among patients with urinary tract infections at a tertiary hospital facility in Alexandria, Egypt: a retrospective record-based classification and regression tree analysis
Source: BMC Infect Dis. 2024 Feb 22;24:246. doi: 10.1186/s12879-024-09086-6 (PMC10885625; doi:10.1186/s12879-024-09086-6)
Supplement: Supplementary file 2 — Supplementary Material 2 [file 12879_2024_9086_MOESM2_ESM.docx]

STROBE Statement—Checklist of items that should be included in reports of ***cohort studies***

|  | **Item No** | **Recommendation** | **Page number/Table/Figure manuscript** |
| --- | --- | --- | --- |
| **Title and abstract** | 1 | (*a*) Indicate the study’s design with a commonly used term in the title or the abstract | Page1(title)  Page2(materials and methods in abstract) |
|  |  | (*b*) Provide in the abstract an informative and balanced summary of what was done and what was found | Page2(abstract) |

| **Introduction** | | |  |
| --- | --- | --- | --- |
| Background/rationale | 2 | Explain the scientific background and rationale for the investigation being reported | Background: page4  Rational: page 5-6 |
| Objectives | 3 | State specific objectives, including any prespecified hypotheses | Page6 last paragraph |
| **Methods** | | |  |
| Study design | 4 | Present key elements of study design early in the paper | Page7 first paragraph |
| Setting | 5 | Describe the setting, locations, and relevant dates, including periods of recruitment, exposure, follow-up, and data collection | Page7 first, third ,and fourth paragraphs |
| Participants | 6 | (*a*) Give the eligibility criteria, and the sources and methods of selection of participants. Describe methods of follow-up | Page7 third paragraph |
|  |  | (*b*) For matched studies, give matching criteria and number of exposed and unexposed | Not applicable |
| Variables | 7 | Clearly define all outcomes, exposures, predictors, potential confounders, and effect modifiers. Give diagnostic criteria, if applicable | Page8,9 |
| Data sources/ measurement | 8* | For each variable of interest, give sources of data and details of methods of assessment (measurement). Describe comparability of assessment methods if there is more than one group | Page9 last paragraph,10,11 |
| Bias | 9 | Describe any efforts to address potential sources of bias | Page13 paragraph 3 |
| Study size | 10 | Explain how the study size was arrived at | Page7 second paragraph |
| Quantitative variables | 11 | Explain how quantitative variables were handled in the analyses. If applicable, describe which groupings were chosen and why | Page11 statistical analysis |
| Statistical methods | 12 | (*a*) Describe all statistical methods, including those used to control for confounding | Page11,12,13 |
|  |  | (*b*) Describe any methods used to examine subgroups and interactions | Not performed |
|  |  | (*c*) Explain how missing data were addressed | Not applicable |
|  |  | (*d*) If applicable, explain how loss to follow-up was addressed | Not applicable |
|  |  | (*e*) Describe any sensitivity analyses | Not performed |
| **Results** | | |  |
| Participants | 13* | (a) Report numbers of individuals at each stage of study—eg numbers potentially eligible, examined for eligibility, confirmed eligible, included in the study, completing follow-up, and analysed | Page13 results |
|  |  | (b) Give reasons for non-participation at each stage | No missing data |
|  |  | (c) Consider use of a flow diagram | Figure1 |
| Descriptive data | 14* | (a) Give characteristics of study participants (eg demographic, clinical, social) and information on exposures and potential confounders | Table1 |
|  |  | (b) Indicate number of participants with missing data for each variable of interest | None |
|  |  | (c) Summarise follow-up time (eg, average and total amount) | Page13,14 |
| Outcome data | 15* | Report numbers of outcome events or summary measures over time | Page14 first paragraph |
| Main results | 16 | (*a*) Give unadjusted estimates and, if applicable, confounder-adjusted estimates and their precision (eg, 95% confidence interval). Make clear which confounders were adjusted for and why they were included | Table3 |
|  |  | (*b*) Report category boundaries when continuous variables were categorized | Statistical methods and footnotes below tables |
|  |  | (*c*) If relevant, consider translating estimates of relative risk into absolute risk for a meaningful time period | Table3 |
| Other analyses | 17 | Report other analyses done—eg analyses of subgroups and interactions, and sensitivity analyses | Not performed |
| **Discussion** | | |  |
| Key results | 18 | Summarise key results with reference to study objectives | Page 19 |
| Limitations | 19 | Discuss limitations of the study, taking into account sources of potential bias or imprecision. Discuss both direction and magnitude of any potential bias | Page23 second paragraph |
| Interpretation | 20 | Give a cautious overall interpretation of results considering objectives, limitations, multiplicity of analyses, results from similar studies, and other relevant evidence | From 19 to 22 |
| Generalisability | 21 | Discuss the generalisability (external validity) of the study results | Page23 first paragraph |
| **Other information** | | |  |
| Funding | 22 | Give the source of funding and the role of the funders for the present study and, if applicable, for the original study on which the present article is based | Not applicable |
